# R77Q and Q3R HIV1-VPR mutations in an otherwise asymptomatic 5-year-old child with repeated ear infections

**DOI:** 10.1099/jmmcr.0.002709

**Published:** 2014-12-01

**Authors:** Rui Soares, Graça Rocha, Célia Nogueira, António Meliço-Silvestre, Teresa Gonçalves

**Affiliations:** ^1^​ Faculty of Medicine, University of Coimbra, Coimbra, Portugal; ^2^​ Center for Neuroscience and Cell Biology, University of Coimbra, Coimbra, Portugal; ^3^​ Hospital Pediátrico de Coimbra, Centro Hospitalar e Universitário de Coimbra, Coimbra, Portugal; ^4^​ Centro Hospitalar e Universitário de Coimbra, Coimbra, Portugal

**Keywords:** asymptomatic, AIDS/HIV-1, HAART, long-term non-progressor, Vpr mutation

## Abstract

**Introduction:**

Viral protein R (Vpr) of human immunodeficiency virus type 1 (HIV-1) has been described as being involved in the progression of AIDS, and specific mutations are associated with long-term non-progressor patients.

**Case presentation:**

We describe the case of a child with repeated ear infections who was otherwise healthy. The patient, a 5-year-old boy, was HIV-1 positive and the viral load at admission was 1 073 899 RNA copies ml^−1^ and 0 % CD4^+^ lymphocytes. A detailed study of the *vpr* gene sequence of the child revealed mutations leading to amino acid substitutions at positions 3 and 77.

**Conclusion:**

The case reported provides clinical support of previous findings that show that the R77Q and Q3R HIV-1 Vpr variants are associated with patients with delayed disease progression.

## Introduction

According to the CDC’s Guide for HIV/AIDS Clinical Care (CDC, 2014), the most common presenting features of human immunodeficiency virus (HIV)-infected children include persistent generalized lymphadenopathy, hepatosplenomegaly, chronic/recurrent diarrhoea, poor growth and fever. A published study showed that in the UK 15–20 % of untreated children will present an AIDS-defining illness by 12 months, with *Pneumocystis* pneumonia at approximately 3–4 months of age. Seventy per cent of perinatal-infected children will exhibit some signs or symptoms by 12 months, and without treatment, the median age of progression to AIDS is approximately 6 years (Hoaré, 2003). More recently, Le Doaré *et al.* (2012) reviewed aspects related to neurodevelopment in HIV-infected infants and children, describing that motor and cognitive parameters, such as language, memory and behaviour, might be modified in these groups of patients.

The HIV type 1 (HIV-1) viral protein R (Vpr) is a 96 aa accessory protein, reported to exhibit numerous biological activities, including modulation of transcription of the viral genome (Sawaya *et al.*, 2000; Romani & Engelbrecht, 2009), induction of T-cell apoptosis and defects in mitosis (Roshal *et al.*, 2001; Chang *et al.*, 2004; Sörgel *et al.*, 2012), nuclear transport of the HIV-1 pre-integration complex (Vodicka *et al.*, 1998), facilitation of reverse transcription (Rogel *et al.*, 1995) and reduction of the HIV mutation rate (Jowett *et al.*, 1999). Vpr has been shown to mediate many processes that contribute to HIV-1 infection, evasion of the immune system and persistence in the host, thus having a role in the morbidity and mortality of AIDS (Kogan & Rappaport, 2011). Vpr is found in HIV-1 virions in infected cells but has also been shown to be present in sera and cerebrospinal fluid of AIDS patients, suggesting that it takes part in several mechanisms of HIV-1 biology (Jacquot *et al.*, 2009) and, as it crosses cell membranes (Henklein *et al.*, 2000), may exert biological effects on different and separate host cells.

Numerous studies have demonstrated that introduction of certain HIV-1 Vpr mutations can dramatically affect its known functions (Di Marzio *et al.*, 1995; Yao *et al.*, 1995; Chen *et al.*, 1999). Mutations such as R73A, R77A and R80A abolish the ability of Vpr to induce apoptosis in T-lymphocytes (Jacotot *et al.*, 2000). Among long-term non-progressors (LTNPs), Lum *et al.* (2003) found a high frequency of the R77Q mutation, and this Vpr mutation impaired apoptosis both *in vivo* and *in vitro*. The Q3R mutation occurs at the N terminus of Vpr. This mutation affects the cytopathic and apoptotic ability of the virus (Somasundaran *et al.*, 2002).

In this work, we describe a clinical case of an HIV-1-positive child who was infected vertically by his asymptomatic mother, either during birth or through breastfeeding. We analysed the *vpr* gene and found that the virus carried mutations in the *vpr* gene leading to amino acid substitutions at positions 77 and 3.

## Case report

In July 2010, a five-year-old boy was admitted to Surgery at the Pediatric Hospital of Coimbra for tonsillectomy and adenoidectomy. He had a medical history of recurrent otological suppurative infections, starting 2 years before, with two or three episodes per month, and antibiotic therapy was frequent. Recurrent epistaxis was reported in the previous 3 weeks before this evaluation. The mother described a period of 4 months of prostration and dysarthric language.

The child was born in Portugal from Portuguese parents, and was attending a pre-school institution. He was born by normal vaginal delivery, without complications, after a full-term gestation. His mother received prenatal care, with pre-eclampsia; HIV serology was negative in the first and last trimesters of pregnancy. At birth, the patient weighed 3460 g and measured 51 cm with an APGAR score of 10 (5 min). The child was breastfed for 2 years. He received all vaccines included in the Portuguese national vaccination programme, including BCG, plus Prevenar (Pfizer) and Meningitec (Pfizer), with no report of complications. At the age of 3 years, he was at the fifth percentile (P50) of weight according to the growth charts. At hospital admission, aged 5 years, he was at P25 for weight and P50 for height (Table 1). On physical examination, he was alert and interactive, with normal mental status, appearing well. The remaining physical clinical examination was normal, except for abdominal distension. On the pre-operative tests, the prothrombin time (15 s; control 1 s) was increased and thromboplastin was normal. A full blood count showed 4.42×10^3^ white blood cells μl^−1^, with an absolute neutrophil count of 1200 and an absolute lymphocyte count of 2700. Serum electrolytes, creatinine and creatine phosphokinase values were normal, but alanine aminotransferase/aspartate aminotransferase levels were elevated to 1939/637 U l-1. Epstein–Barr virus serology was IgM positive, but all other hepatotropic virus markers were negative. A 0 % CD4^+^ lymphocyte value prompted the clinicians to test for HIV, revealing a positive result with an HIV viral load of 1 073 899 RNA copies ml^−1^ (6.03 log) (Table 1). According to the CDC staging system, at this point the HIV clinical disease staging of the boy could be classified as A3. After the diagnosis of HIV infection in the child, his parents also tested positive for HIV-1. As the child’s mother seroconverted after the 32 week of pregnancy, it is impossible to determine whether the child was infected peripartum or during breastfeeding. The mother was considered asymptomatic, and still remains as such, although at the child’s diagnosis it could not be considered a LTNP, as he was infected for less than 7 years (Poropatich & Sullivan, 2011).

Upon diagnosis, he started a combined oral therapy of lamivudine 4 mg kg^−1^ twice a day, zidovudine 180 mg m^−2^ twice a day, lopinavir/ritonavir 10/2.5 mg^−1^ kg^−1^ twice a day. The response to treatment was excellent, as assessed by the increasing CD4^+^ percentage and the decreasing plasma viral load (Table 1). The immunological recovery was achieved without any clinical manifestations suggestive of immune reconstitution inflammatory syndrome. One month after treatment was started, the aminotransferase value returned to normal. After 8 months, the plasma HIV RNA load was undetectable and the CD4^+^ level increased to 17.8 % with a CD4^+^/CD8^+^ ratio of 0.3. Eighteen months after treatment, the CD4^+^ level reached to 34.7 % and the CD4^+^/CD8^+^ ratio was 0.63. At this stage, the clinical examination was normal, without symptoms of disease (Table 1). The mother never developed any signs of disease compatibly with AIDS, while the father rapidly progressed to AIDS (results not shown).

As part of an ongoing surveillance programme of LTNPs, we searched in the child and parents for genetic variants of the *vpr* gene. For this, leukocytes were extracted from the patient’s blood using a Ficoll (Lymphoprep; Axis-Shield) gradient methodology, based on the manufacturer’s instructions. Leukocyte DNA extraction was performed using a MagNA Pure Compact with a MagNA Pure Compact Nucleic Acid Isolation kit I (Roche). Amplification of the *vpr* gene was performed as described by Zhao *et al.* (2002), with slight modifications. Briefly, two sets of primers were designed and used in two successive amplifications: the external primers, Fwd-Vpr (5'-GGAAAACAGATGGCAGGTGATG-3') and Rev-Vpr (5'-TCTCCGCTTCTTCCTGCCAT-3') and the internal primers Vpr1f (5'-ATAGTTAGTCCTAGGTGTGA-3') and Nint2 (5'-TTCCTGGATGCTTCCAGGGCTCTA-3'). For amplification, we used a Phusion Hot Start High-Fidelity DNA polymerase (Finnzymes). The amplicon was purified and sequenced elsewhere (LGC Genomics). The sequences obtained were analysed with the mega4 program (http://www.megasoftware.net/mega4/mega.html), to search for mutations. We found that the child’s HIV-1 *vpr* gene had point mutations, some of which resulted in codon changes (see Fig. S1, available in the online Supplementary Material). The predicted resulting amino acid sequence led to an amino acid change at position 77, from arginine to glutamine (R77Q), and at position 3 from glutamine to arginine (Q3R). The *vpr* gene of the parents revealed a predicted change at position 77, but the codons indicated a change from arginine to histidine (R77H) (Fig. S1).

## Discussion

In healthcare settings, the HIV diagnosis is easier in children born from a previously HIV-positive diagnosed mother. Increased difficulties arise when the mother was not previously identified as HIV positive. In the clinical case now reported by us, a child had a clinical history of repeated ear infection, which was never associated with HIV infection due to an unsuspected and asymptomatic mother. This recurrence prompted the child to undergo surgical intervention, and during routine analysis some haematological/biochemical indicators unveiled the suspicion of HIV infection.

In fact, ear-related and repeated symptoms have been described as one of the first signs of HIV infection. Children with HIV infection may have their primary presentation to an otorhinolaryngologist, who should have appropriate thresholds for suspecting the diagnosis (Hoaré, 2003). Ear infections are common in childhood, and so only the presence of other signs or symptoms together with a clinical/familial history should drive the physician to an HIV diagnosis. In the case described here, another sign/symptom was present in the child: a prolonged period of prostration and of dysarthric language. Language impairments have been indicated as a prominent cognitive feature of HIV infection in children (Wolters *et al.*, 1997), with ataxic dysarthria being described as one of these disorders (Lopez *et al.*, 1994). The asthenia described in the child could be related to central nervous system involvement, as described by Woods *et al.* (2010).

Although numerous studies have demonstrated that HIV-1 Vpr mutations could dramatically affect its known functions (Di Marzio *et al.*, 1995; Yao *et al.*, 1995; Chen *et al.*, 1999), these mutations were artificially manipulated and probably did not represent the profile of the naturally occurring mutations over the course of the infection. Studies in isolated Vpr from HIV-1-infected patients are of crucial importance to correlate naturally occurring mutations in this protein and its role in disease progression. An initial study by Lum *et al.* (2003) demonstrated the presence of a point mutation at position 77 of HIV-1 Vpr that led to an R77Q substitution with a higher frequency (80 %) in Vpr alleles coming from LTNPs compared with patients developing progressive disease (Lum *et al.*, 2003). This was confirmed by Mologni *et al.* (2006), while others were not able to find any association between the R77Q substitution and disease progression (Fischer *et al.*, 2004; Rajan *et al.*, 2006). With the case reported here, more clinical evidence of this association is added, as we have described a Vpr R77Q substitution in a mildly symptomatic/asymptomatic HIV-infected child, where all the major HIV-related diseases were absent. Moreover, the Q3R mutation was also present, and this mutation has been described previously in an LTNP patient (Somasundaran *et al.*, 2002) due to low cytopathic effect. Andersen *et al.* (2008) showed that both the R77Q and Q3R mutants induced G2 arrest but with a lower level of apoptosis when compared with wild-type Vpr.

The vaccination of infected children has generated some discussion, as the immune response is not always a disturbing factor, as one might expect. A study by Bickel *et al.* (2013) showed that low vitamin D levels were correlated with a weak immune response in HIV-infected patients. On the other hand, other reports have demonstrated that vaccination in HIV-infected patients could work like a pump in the immune response (Mphahlele & Mda 2012). In the clinical case reported here, the child had no adverse reactions to vaccination and no signs of inefficient immunization against the common pathogens in pre-school children.

The report of asymptomatic HIV-1 carriers is becoming more frequent (Poropatich & Sullivan, 2011; Gaardbo *et al.*, 2012). However, the rationale underlying this novel characteristic of HIV infection has not yet been established. Is it due to a more adapted immune response of the host or to a less virulent agent? The clinical case here reported is an example of a patient who could be considered in the asymptomatic category A3, with the pathologies usually found in non-infected children, together with – at least at hospital admission – a zero count of CD4^+^ cells and an extremely high viral load. Except for the ear infections and the mother’s information of prostration and dysarthric language, no major pathologies were found. Moreover, the child’s mother has never developed any sign of disease compatible with AIDS. Another important issue associated with this case is the efficiency of therapy, which rapidly normalized the CD4^+^ count and decreased the viral load to undetectable levels. We believe that the clinical case here reported presents some unique issues: (i) no serious opportunistic infections in an HIV-1-infected child attending a pre-school institution; (ii) no adverse reactions to vaccination; (iii) an asymptomatic mother; (iv) rapid immune recovery and plasma viral load decreases to undetectable levels; and (v) the presence of two Vpr mutations, at positions 3 and 77.

**Table 1.** Clinical and laboratory data

Characteristic/treatment	Day 1*	June 2010	July 2010	December 2010	October 2011	April 2012
Age	5 years, 1 month	5 years, 3 month	5 years, 4 months	5 years, 9 months	6 years, 7 months	7 years, 1 month
Weight (kg)	16	16.6	17.8	18.8	20.2	22
Percentile	25/50	25/50	25/50	25/50	25/50	25
HIV RNA copies ml^−1^	1 073 899	680	123	<20	<20	<20
CD4^+^ T-cells ml^−1^	0	0.07	0.1	672	1310	1553
CD4^+^ T-cells (%)	<1	2.1	2.9	17.8	34.7	41.1
CD4^+^/CD8^+^ ratio	–	–	–	0.3	0.63	0.75
**Antiretroviral medication (mg)**†						
Months	0	2	3	8	18	24
Zidovudine	130	130	150	150	150	150
Lamivudine	70	70	75	75	75	75
Lopinavir/ritonavir	160/20	160/20	200/25	200/25	200/25	200/25

<ast?> * Day 1 was 6 days after admission.

**<dagger?>:** † Twice daily dose.

## Abbreviations

HIV-1: human immunodeficiency virus type 1
LTNP: long-term non-progressor.

## References

[jmmcr002709-Andersen1] Andersen J.L, Le Rouzic E, Planelles V (2008). HIV-1 Vpr: mechanisms of G2 arrest and apoptosis. Exp Mol Pathol.

[jmmcr002709-Bickel1] Bickel M, Lassmann C, Wieters I, Doerr H.W, Herrmann E, Wicker S, Brodt H.R, Stephan C, Allwinn R, Jung O (2013). Immune response after a single dose of the 2010/11 trivalent, seasonal influenza vaccine in HIV-1-infected patients and healthy controls. HIV Clin Trials.

[jmmcr002709-CDC1] CDC (2014). http://aidsetc.org/guide/contents.

[jmmcr002709-Chang1] Chang F, Re F, Sebastian S, Sazer S, Luban J (2004). HIV-1 Vpr induces defects in mitosis, cytokinesis, nuclear structure, and centrosomes. Mol Biol Cell.

[jmmcr002709-Chen1] Chen M, Elder R.T, Yu M, O’Gorman M.G, Selig L, Benarous R, Yamamoto A, Zhao Y (1999). Mutational analysis of Vpr-induced G2 arrest, nuclear localization, and cell death in fission yeast. J Virol.

[jmmcr002709-DiMarzio1] Di Marzio P, Choe S, Ebright M, Knoblauch R, Landau N.R (1995). Mutational analysis of cell cycle arrest, nuclear localization and virion packaging of human immunodeficiency virus type 1 Vpr. J Virol.

[jmmcr002709-Fischer1] Fischer A, Lejczak C, Lambert C, Roman F, Servais J, Karita E, Allen S, Schmi t.J.C, Arendt V (2004). Is the Vpr R77Q mutation associated with long-term non-progression of HIV infection?. AIDS.

[jmmcr002709-Gaardbo1] Gaardbo J.C, Hartling H.J, Gerstoft J, Nielsen S.D (2012). Thirty years with HIV infection-nonprogression is still puzzling: lessons to be learned from controllers and long-term non-progressors. AIDS Res Treat.

[jmmcr002709-Henklein1] Henklein P, Bruns K, Sherman M.P, Tessmer U, Licha K, Kopp J, de Noronha C.M, Greene W.C, Wray V, Schubert U (2000). Functional and structural characterization of synthetic HIV-1 Vpr that transduces cells, localizes to the nucleus, and induces G2 cell cycle arrest. J Biol Chem.

[jmmcr002709-Hoare1] Hoaré S (2003). HIV infection in children-impact upon ENT doctors. Int J Pediatr Otorhinolaryngol.

[jmmcr002709-Jacotot1] Jacotot E, Ravagnan L, Loeffler M, Ferri K.F, Vieira H.L, Zamzami N, Costantini P, Druillennec S, Hoebeke J, other authors (2000). The HIV-1 viral protein R induces apoptosis via a direct effect on the mitochondrial permeability transition pore. J Exp Med.

[jmmcr002709-Jacquot1] Jacquot G, Le R.E, Maidou-Peindara P, Maizy M, Lefrere J.J, Daneluzzi V, Monteiro-Filho C.M.R, Hong D, Planelles V, Morand-Joubert L, Benichou S (2009). Characterization of the molecular determinants of primary HIV-1 Vpr proteins: impact of the Q65R and R77Q substitutions on Vpr functions. PLoS One.

[jmmcr002709-Jowett1] Jowett J.B, Xie Y.M, Chen I.S (1999). The presence of human immunodeficiency virus type 1 Vpr correlates with a decrease in the frequency of mutations in a plasmid shuttle vector. J Virol.

[jmmcr002709-Kogan1] Kogan M, Rappaport J (2011). HIV-1 accessory protein Vpr: relevance in the pathogenesis of HIV and potential for therapeutic intervention. Retrovirology.

[jmmcr002709-LeDoare1] Le Doaré K, Bland R, Newell M.L (2012). Neurodevelopment in children born to HIV-infected mothers by infection and treatment status. Pediatrics.

[jmmcr002709-Lopez1] Lopez O.L, Becker J.T, Dew M.A, Banks G, Dorst S.K, McNeil M (1994). Speech motor control disorder after HIV infection. Neurology.

[jmmcr002709-Lum1] Lum J.J, Cohen O.J, Nie Z, Weaver J.G, Gomez T.S, Yao X.J, Lynch D, Pilon A.A, Hawley N, other authors (2003). Vpr R77Q is associated with long-term nonprogressive HIV infection and impaired induction of apoptosis. J Clin Invest.

[jmmcr002709-Mologni1] Mologni D, Citterio P, Menzaghi B, Zanone P.B, Riva C, Broggini V, Sinicco A, Milazzo L, Adorni F, other authors (2006). Vpr and HIV-1 disease progression: R77Q mutation is associated with long-term control of HIV-1 infection in different groups of patients. AIDS.

[jmmcr002709-Mphahlele1] Mphahlele M.J, Mda S (2012). Immunising the HIV-infected child: a view from sub-Saharan Africa. Vaccine.

[jmmcr002709-Poropatich1] Poropatich K, Sullivan D.J (2011). Human immunodeficiency virus type 1 long-term non-progressors: the viral, genetic and immunological basis for disease non-progression. J Gen Virol.

[jmmcr002709-Rajan1] Rajan D, Wildum S, Rucker E, Schindler M, Kirchhoff F (2006). Effect of R77Q, R77A and R80A changes in Vpr on HIV-1 replication and CD4 T cell depletion in human lymphoid tissue ex vivo. AIDS.

[jmmcr002709-Rogel1] Rogel M.E, Wu L.I, Emerman M (1995). The human immunodeficiency virus type 1 *vpr* gene prevents cell proliferation during chronic infection. J Virol.

[jmmcr002709-Romani1] Romani B, Engelbrecht S (2009). Human Immunodeficiency virus type 1 Vpr: functions and molecular interactions. J Gen Virol.

[jmmcr002709-Roshal1] Roshal M, Zhu Y, Planelles V (2001). Apoptosis in AIDS. Apoptosis.

[jmmcr002709-Sawaya1] Sawaya B.E, Khalili K, Gordon J, Taube R, Amini S (2000). Cooperative interaction between HIV-1 regulatory proteins Tat and Vpr modulates transcription of the viral genome. J Biol Chem.

[jmmcr002709-Somasundaran1] Somasundaran M, Sharkey M, Brichacek B, Luzuriaga K, Emerman M, Sullivan J.L, Stevenson M (2002). Evidence for a cyto-pathogenicity determinant in HIV-1 Vpr. Proc Natl Acad Sci U S A.

[jmmcr002709-Sorgel1] Sörgel S, Fraedrich K, Votteler J, Thomas M, Stamminger T, Schubert U (2012). Perinuclear localization of the HIV-1 regulatory protein Vpr is important for induction of G2-arrest. Virology.

[jmmcr002709-Vodicka1] Vodicka M.A, Koepp D.M, Silver P.A, Emerman M (1998). HIV-1 Vpr interacts with the nuclear transport pathway to promote macrophage infection. Genes Dev.

[jmmcr002709-Wolters1] Wolters P.L, Brouwers P, Civitello L, Moss H.A (1997). Receptive and expressive language function of children with symptomatic HIV infection and relationship with disease parameters: a longitudinal 24-month follow-up study. AIDS.

[jmmcr002709-Woods1] Woods S.P, Iudicello J.E, Dawson M.S, Weber E, Grant I, Letendre S.L, HIV Neurobehavioral Research Center (HNRC) Group (2010). HIV-associated deficits in action (verb) generation may reflect astrocytosis. J Clin Exp Neuropsychol.

[jmmcr002709-Yao1] Yao X.J, Subbramanian R.A, Rougeau N, Boisvert F, Bergeron D, Cohen E.A (1995). Mutagenic analysis of human immunodeficiency virus type 1 Vpr: role of a predicted N-terminal α-helical structure in Vpr nuclear localization and virion incorporation. J Virol.

[jmmcr002709-Zhao1] Zhao Y, Chen M, Wang B, Yang J, Elder R.T, Song X.Q, Yu M, Saksena N.K (2002). Functional conservation of HIV-1 Vpr and variability in a mother–child pair of long-term non-progressors. Virus Res.

